# The Peritoneal Cancer Index is a Strong Predictor of Incomplete Cytoreductive Surgery in Ovarian Cancer

**DOI:** 10.1245/s10434-020-08649-6

**Published:** 2020-05-29

**Authors:** Björg Jónsdóttir, Marta Lomnytska, Inger Sundström Poromaa, Ilvars Silins, Karin Stålberg

**Affiliations:** 1 Department of Women’s and Children’s Health, Uppsala University, Uppsala, Sweden; 2 Department of Obstetrics and Gynecology, Uppsala University Hospital, Uppsala, Sweden; 3 Institute of Oncology and Pathology, Karolinska Institutet, Karolinska, Stockholm, Sweden

## Abstract

**Background:**

Extent of tumor load is an important factor in the selection of ovarian cancer patients for cytoreductive surgery (CRS). The Peritoneal Cancer Index (PCI) gives exact information on tumor load but still is not standard in ovarian cancer surgery. The aim of this study was to find a PCI cutoff for incomplete CRS. The secondary aims were to identify reasons for open-close surgery and to compare surgical complications in relation to tumor burden.

**Methods:**

The study included 167 women with stage III or IV ovarian cancer scheduled for CRS. Possible predictors of incomplete surgery were evaluated with receiver operator curves, and a PCI cutoff was identified. Surgical complications were analyzed by one-way analysis of variance and Chi square tests.

**Results:**

The median PCI score for all the patients was 22 (range 3–37) but 33 (range 25–37) for the patients with incomplete surgery (*n* = 19). The PCI predicted incomplete CRS, with an area under the curve of 0.94 (95% confidence interval [CI], 0.91–0.98). Complete CRS was obtained for 67.2% of the patients with a PCI higher than 24, who experienced an increased rate of complications (*p* = 0.008). Overall major complications were found in 16.9% of the cases. Only 28.6% of the patients with a PCI higher than 33 achieved complete CRS. The reason for open-close surgery (*n* = 14) was massive carcinomatosis on the small bowel in all cases.

**Conclusion:**

The study found PCI to be an excellent predictor of incomplete CRS. Due to a lower surgical success rate, the authors suggest that neoadjuvant chemotherapy could be considered if the PCI is higher than 24. Preoperative radiologic assessment should focus on total tumor burden and not necessarily on specific regions.

**Electronic supplementary material:**

The online version of this article (10.1245/s10434-020-08649-6) contains supplementary material, which is available to authorized users.

Ovarian cancer, the fourth most common cause of cancer-related death among women, is diagnosed for 240,000 women globally every year.^1^ The 5-year survival rate in Sweden has been increasing in recent years and currently is 49%.^2^ At diagnosis, the majority of patients have stages III or IV disease according to the International Federation of Gynecology and Obstetrics (FIGO) staging system,^3^ meaning that the tumor has disseminated into the peritoneal cavity and its organs.^4^

The mainstay of treatment is cytoreductive surgery (CRS), with the aim of removing all macroscopic tumors because the absence of any residual disease is the most important factor for survival and prognosis.^5^ Massive ascites production, poor nutrition status, and significant pleural effusion usually are associated with extensive disease spread, in which case, the patient is assigned to neoadjuvant chemotherapy (NACT). Furthermore, NACT candidates also are patients with nonresectable carcinomatosis, sometimes determined after laparotomy (open-close surgery). Consequently, identifying these patients preoperatively is of great value.^6^

Currently, the extent of tumor load is estimated by different imaging methods, usually by computed tomography (CT) and magnetic resonance imaging (MRI) or preoperative laparoscopy. However, no universally accepted reference standard exists for the imaging of peritoneal carcinomatosis.^7^

To describe peritoneal carcinomatosis, the Peritoneal Cancer Index (PCI) was introduced by Jacquet and Sugarbaker^8^ initially for carcinomatosis of colorectal cancer and mesothelioma. In colorectal cancer, PCI is the most important prognostic factor, showing a linear relationship with overall survival.^9^ A consensus on a cutoff value for treatment has not been clearly established. However, surgery is not recommended for patients who have colorectal carcinomatosis, with a PCI higher than 20.^10^ In ovarian cancer, assessment of PCI still is not a standard of care in clinical practice or in surgical studies.

Different cutoffs of total PCI have been applied for the investigation of resectability and survival in ovarian cancer. For these outcomes, most studies have used PCI cutoffs of 10–15.^11^^–^13 Furthermore, it has been suggested that instead of the total PCI score, selected PCI regions, such as the small intestine and the hepatoduodenal ligaments, are better predictors for resectability and survival.^14^ Unfortunately, these areas are difficult to assess on preoperative imaging, especially regarding diffuse carcinomatosis on the intestines.^15^

If information on tumor load from PCI were adequately estimated preoperatively, it could be used in selecting patients for primary surgery. In an attempt to achieve this, the PCI has been used in the interpretation of images.^16^^–^18 Currently, it is not known whether the PCI cutoff score for colorectal cancer can also be applied for ovarian cancer.

Establishment of a PCI cutoff for CRS in ovarian cancer would be of great value for identifying inoperable patients. This primary aim of this study was to find a PCI cutoff for incomplete CRS. The secondary aims were to identify reasons for open-close surgery and to compare surgical complications in relation to tumor burden.

## Materials and Methods

This single-center study analyzed patients with ovarian, fallopian tube, or primary peritoneal cancer (later referred to as ovarian cancer) treated at Uppsala University hospital, an ESGO-certified tertiary referral center that performs 70–80 primary ovarian cancer operations per year.

The preoperative workup included diagnosis based on histology and/or on cytology and a tumor marker profile suggesting gynecologic cancer. A CT scan of the abdomen, pelvis, and thorax (chest) was an obligatory part of the workup. Patients were selected for neoadjuvant chemotherapy (NACT) on the basis of poor general condition, advanced age, massive ascites, hypoalbuminemia, significant pleural effusion, or other factors indicating massive disease spread (e.g., major carcinomatosis on the CT scan).

The inclusion criteria specified women who had suspected ovarian cancer with carcinomatosis, recorded data on estimated PCI during surgery, and disease in the range of FIGO stages IIIB to IVB. Women who previously had undergone open-close surgery and received NACT were not included in the study.

Data on FIGO stage, histology, patient characteristics, surgery, and surgical complications were collected from the medical records. A certified gynecologic cancer surgeon participated in all the operations. The study was approved by the Swedish Ethical Review Board (Dnr 2018/071, Dnr 2019/05513).

### Peritoneal Cancer Index and Cytoreduction Score

Surgery was performed with the intention of achieving complete cytoreduction. At the beginning of the surgery, a PCI score was estimated as follows. For the anatomic distribution, 13 regions were defined. Two transverse planes and two sagittal planes divide the abdominopelvic cavity into nine regions. Regions 9–12 divide the small bowel. Lesion size (LS) refers to the greatest diameter of the tumor implants distributed on the peritoneal surfaces, with LS ranging from 0 (no tumor seen) to 3 (tumor > 5 cm). The PCI score ranges from 0 to 39 (Supplementary Data 1).^8^

At completion of the surgery, the completeness of the cytoreduction score (CCS) was estimated as follows: CC0 (no residual disease), CC1 (residual nodules < 2.5 mm), CC2 (residual nodules between 2.5 mm and 2.5 cm), and CC3 (residual nodules > 2.5 cm). Complete cytoreduction was defined as CC0 and CC1, whereas CC2 and CC3 were considered to indicate incomplete cytoreduction. If the surgeon did not see any possibility for radical or near radical surgery, no surgery was performed, the abdomen was closed (open-close procedure), and the procedure was classified as incomplete cytoreduction.

To evaluate the complexity of operations, the Aletti score was used, which divided the operations into three groups using low, intermediate, or high complexity scores. These scores are based on the number of procedures performed in every operation and their degree of difficulty.^19^

The 30-day postoperative complications were graded with the Clavien–Dindo classification system.^20^

### Statistical Analyses

The PCI cutoff values for incomplete CRS were evaluated with a receiver operator characteristic (ROC) curve and other possible predictors of incomplete CRS explored with ROC curves. The predictors tested were age, preoperative albumin levels, body mass index, smoking, American Society of Anesthesiologists [ASA] score, physical status classification system, FIGO stage, treatment, histology, and PCI score. Subgroups identified by PCI cutoff were compared regarding peri- and postoperative complications using an independent *t* test, Chi square tests, or one-way analysis of variance (ANOVA). Patient characteristics, divided into groups by completion of CRS, were compared with an independent *t* test and Chi square tests. A two-sided *p* value lower than 0.05 was considered statistically significant. The data were analyzed using IBM SPSS statistics 24 (IBM Corp.,  Armonk,  NY, USA).

## Results

**Fig. 1:**
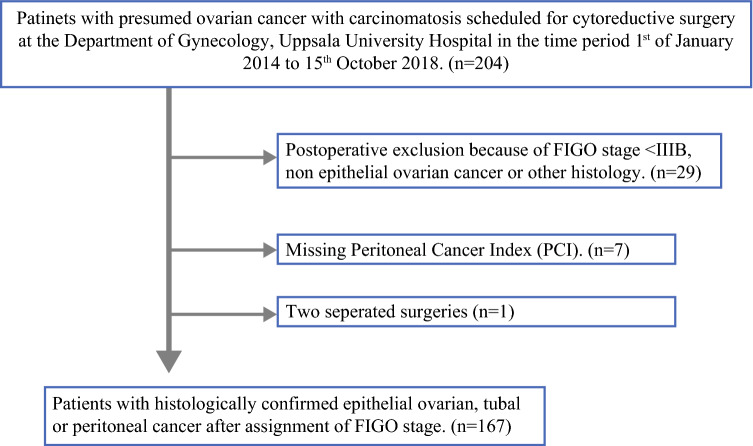
Flowchart showing recruitment of the study population

The study enrolled 204 women scheduled for primary or interval CRS from 1 January 2014 to 15 October 2018 at Uppsala University Hospital. The study excluded 29 women due to a FIGO stage lower than IIIB or because of histology types other than epithelial ovarian, fallopian tube, or primary peritoneal cancer. Seven women were excluded due to missing registration of PCI (Fig. 1). Thus, 167 women were included in the study. None of the women had undergone preoperative laparoscopy.

Clinical variables and tumor characteristics of the study population are described in Table 1. The majority of the patients had high-grade serous adenocarcinoma and FIGO stage IIIC or IV disease, and 48.5% had received neoadjuvant chemotherapy (NACT).

**Table 1:** Patient and tumor characteristics in the entire study population and in relation to completeness of cytoreductive surgery (CRS)^a^

	All patients (*n* = 167) *n* (%)	Complete CRS (*n* = 148) *n* (%)	Incomplete CRS (*n* = 19)^b^ *n* (%)	*p* value
Age: years (IQR)	64 (55–71)	65 (55–71)	59 (55–65)	0.3
Albumin: g/l (IQR)	35.0 (32.0–37.0)	35.0 (32.0–37.0)	33.0 (32.0–36.0)	0.7
BMI (kg/m^2^)				0.07
< 20.0	1 (0.6)	1 (0.7)	0	
20.0–25.0	86 (51.8)	72 (49.0)	14 (73.7)	
26.0–30.0	41 (24.7)	36 (24.5)	5 (26.3)	
> 30.0	38 (22.9)	38 (25.9)	0	
Smoking				0.3
No	141 (85.5)	125 (84.5)	16 (94.1	
Yes	24 (14.5)	23 (15.5)	1 (5.9)	
ASA score				0.7
10	10 (6.1)	10 (6.8)	0	
20	108 (65.5)	95 (64.6)	13 (72.2)	
30	46 (27.9)	41 (27.9)	5 (27.8)	
40	1 (0.6)	1 (0.7)	0 (0)	
FIGO stage				0.6
IIIB	2 (1.2)	2 (1.4)	0	
IIIC	86 (51.5)	78 (52.7)	8 (42.1)	
IV	79 (47.3)	68 (45.9)	11 (57.9)	
Primary treatment				0.3
Primary surgery	86 (51.5)	74 (50.0)	12 (63.2)	
NACT	81 (48.5)	74 (50.0)	7 (36.8)	
Histology				0.9
HGSC	140 (83.8)	123 (83.1)	17 (89.5)	
Endometrioid	3 (1.8)	3 (2.0)	0 (0)	
Clear cell	7 (4.2)	6 (4.1)	1 (5.3)	
LGSC	14 (8.4)	13 (8.8)	1 (5.3)	
Carcinosarcoma	3 (1.8)	3 (2.0)	0 (0)	
PCI	22.0 (12.0–27.0)	19.0 (12.0–25.0)	33.0 (30.0–35.0)	< 0.001

IQR, interquartile range; BMI, body mass index; ASA, American Society of Anesthesiology; FIGO, International Federation of Gynecology and Obstetrics; NACT, neoadjuvant chemotherapy; HGSC, high-grade serous adenocarcinoma; LGSC, low-grade serous adenocarcinoma

^a^Percentages are presented in relation to available information. Missing information in 0–6 of variables. Statistics were obtained by independent *t* tests or Chi square tests

^b^Incomplete CRS is defined as Completeness of Cytoreductive Score (CCS) 2 or 3

The median PCI score for the entire study population was 22 (range 3–37; interquartile range [IQR] 12–27). Complete CRS was achieved for 148 patients (88.1%), corresponding to CC0 in 121 cases (82%). Of 19 patients who had incomplete CRS, 14 underwent open-close surgery because of inoperability due to massive carcinomatosis on the small bowel. For the remaining five patients, CRS resulted in CC2-3 despite surgical effort that found carcinomatosis at the end of surgery on the surface of the small bowel or basal pleura or on the main artery of the liver. One patient was judged too weak for completion of the larger surgery because of a preoperative pulmonary embolism.

Patients with an incomplete CRS had a higher median PCI of 33 (range 25–37; IQR 30–35), whereas those with a complete CRS had a median PCI of 19 (range 3–34; IQR 12–25) (*p* < 0.001; Table 1).

**Fig. 2:**
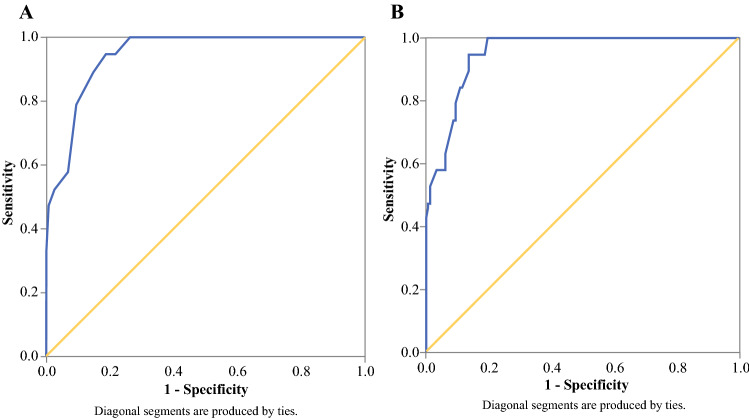
**a** Receiver operator curve (ROC) for the Peritoneal Cancer Index (PCI) regarding incomplete cytoreductive surgery (CRS) (area under the curve [AUC], 0.945; 95% confidence interval [CI], 0.91–0.98). **b** ROC curve for PCI, primary treatment, and body mass index (BMI) regarding incomplete CRS (AUC, 0.953; 95% CI, 0.92–0.99)

Predictors for incomplete CRS were determined by ROCs. According to these analyses, the best predictor of incomplete CRS was the intraoperative PCI score with an AUC of 0.945 (95% confidence interval [CI], 0.91–0.98; Table 2; Fig. 2a). None of the remaining variables could predict incomplete CRS, and all AUCs were lower than 0.6 (Table 2). Furthermore, the addition of primary treatment and body mass index (BMI) to the PCI score minimally improved the predictive value (AUC, 0.953; 95% CI, 0.92–0.98; Fig. 2b).

**Table 2:** Receiver operator curves (ROCs) for possible predictors of incomplete cytoreductive surgery (CRS)

	AUC (95% CI)^a^
Age	0.398 (0.28–0.52)
Albumin	0.416 (0.29–0.55)
BMI	0.348 (0.24–0.46)
Smoking	0.452 (0.32–0.59)
ASA score	0.510 (0.39–0.65)
FIGO stage	0.563 (0.43–0.70)
Primary treatment	0.434 (0.29–0.57)
Histology	0.467 (0.34–0.59)
PCI	0.945 (0.91–0.98)

AUC, area under the curve; CI, confidence interval; BMI, body mass index; ASA, American Society of Anesthesiology; FIGO, International Federation of Gynecology and Obstetrics; PCI, Peritoneal Cancer Index

^a^AUC > 0.9 indicates excellent prediction; AUC 0.8–0.9 indicates good prediction

Based on these analyses, two cutoffs were established. First, a PCI score of 24 or lower had a sensitivity of 100% for detection of cases with incomplete CRS. The specificity of a PCI cutoff score higher than 24 was 73.6% because many patients above this cutoff still remained operable. Overall, 39 patients (67.3%) with a PCI score higher than 24 had a complete CRS. Second, to obtain high specificity for detecting cases of incomplete CRS, a PCI cutoff of 33 was identified, at which the sensitivity and specificity for incomplete CRS cases were respectively 45.0% and 99.3%. Among the patients with a PCI higher than 33, 71.4% had incomplete operations.

In Table 3, the surgical complexity and complications are analyzed by dividing the study population into three groups using the two cutoff levels. No difference regarding histology or FIGO stage was found, although the tendency in the group with a PCI of 33–37 was that fewer patients had received NACT. Overall, the Aletti surgical complexity score (SCS)^19^ was high (≥ 8) for 71.2% of the patients, and the stoma rate was 45.1%, including end ileostomies, loop ileostomies, and colostomies. Major complications (Clavien–Dindo grade 3 or higher) were found in 16.9% of the cases. In one case, the patient died within 30 days after surgery. Other common major complications were postoperative pleural effusion requiring drainage, pneumothorax, sepsis, and reoperations due to anastomosis leakage (*n* = 1), ureter injury (*n* = 1), and bleeding (*n* = 2).

**Table 3:** Peri- and postoperative complications according to Peritoneal Cancer Index (PCI) cutoffs^a^

	PCI 1–24	PCI 25–32	PCI 33–37	*p* value
All patients	(*n* = 109) *n* (%)	(*n* = 44) *n* (%)	(*n* = 14) *n* (%)	
Primary treatment				
Primary surgery	48 (44.0)	27 (61.4)	11 (78.6)	0.016
NACT	61 (56.0)	17 (38.6)	3 (21.4)	
FIGO stage				0.460
IIIB	2 (1.8)	0	0	
IIIC	60 (55.0)	21 (47.7)	5 (35.7)	
IV	47 (43.1)	23 (52.3)	9 (64.3)	
Completeness of surgery				0.000
Complete CRS (CC0-1)	109 (100)	35 (79.5)	4 (28.6)	
Incomplete CRS (CC2-3)	0	9 (20.5)	10 (71.4)	

Surgical patients^a^	(*n* = 109)	(*n* = 37)	(*n* = 7)	
Primary treatment				0.004
Primary surgery	48 (44.0)	24 (64.9)	6 (85.7)	
NACT	61 (56.0)	13 (35.1)	1 (14.3)	
Mean surgery duration: min (range)	282 (110–609)	416 (258–710)	493 (160–823)	0.000
Mean blood loss: ml (range)	481 (20–2000)	635 (200–1500)	821 (250–1500)	0.005
SCS				0.005
Low (< 3)	9 (8.3)	0	0	
Intermediate (4–7)	32 (29.4)	2 (5.4)	1 (14.3)	
High (> 8)	68 (62.4)	35 (94.6)	6 (85.7)	
Clavien–Dindo				0.024
0	63 (57.8)	10 (27.0)	2 (28.6)	
1	0	1 (2.7)	0	
2	33 (30.3)	16 (43.2)	2 (28.6)	
3a	7 (6.4)	6 (16.2)	1 (14.3)	
3b	2 (1.8)	1 (2.7)	0	
4a	3 (2.8)	3 (8.1)	2 (28.6)	
5	1 (0.9)	0	0	
Stoma	33 (30.3)	30 (81.1)	6 (85.7)	0.000
Ileostomy	16 (14.7)	14 (37.8)	4 (57.1)	
Colostomy	17 (15.6)	16 (43.2)	2 (28.6)	

NACT, neoadjuvant chemotherapy; FIGO, International Federation of Gynecology and Obstetrics; CRS, cytoreductive surgery; SCS, surgical complexity score

^a^Statistics were obtained by one-way analysis of variance (ANOVA) or Chi square tests

^a^Inoperable patients (open-close) were excluded

The rate of peri- or postoperative complications was increased in both cutoff groups (*p* = 0.024), with major complications for 27% of the group with a PCI of 25–32 and 42.9% of the group with a PCI of 33–37. The operation time, SCS, and blood loss increased with a higher PCI. The duration of surgery was 1.7 times longer for the group with a PCI of 33–37 than for the group with a PCI of 1–24. The stoma rate was 30.3% for the patients with a PCI lower than 24 compared with 81.1% and 85.7% for the groups with the higher PCI (Table 3).

## Discussion

In our cohort of patients with advanced ovarian, fallopian tube, and peritoneal cancer, the intraoperative PCI score was found to be an excellent predictor of incomplete CRS. All the patients in the study population with incomplete surgery had a PCI higher than 25, and the reason for open-close surgery in all cases was carcinomatosis on the small bowel.

A limitation of our study was that it included few cases with incomplete surgery. Its strengths were the homogeneity of the patients, surgeries at one center performed in a standardized manner by two certified gynecologic cancer surgeons, and a perioperative PCI evaluated at the time of each surgery. However, the homogeneity of the patients also may also be considered a limitation because it makes generalization to other centers more difficult.

Various PCI cutoff values for indicating incomplete CRS have been discussed. Llueca et al.^21^ suggested that ovarian cancer patients with a PCI higher than 20 should be assigned to NACT because of complication risks. Lampe et al.^22^ suggested that complete CRS can be achieved up to a maximum PCI of 25. Older studies analyzing PCI as a predictor of survival have suggested lower cutoff values of 10^11^ and 13,^23^ with worse prognosis associated with a higher PCI. These studies resulted in more extensive surgery. The current study found that complete cytoreduction can be obtained for patients with a PCI up to 34.

Compared with many previous studies, the current study population had a higher tumor load, as indicated by the median PCI of 22, and 45% of the patients had stage IV disease.

At Uppsala University Hospital, surgery is performed for 70–80 primary ovarian cancer patients per year, and a complete CRS rate of 88.1% has been achieved, which is comparable with the rates in large international centers.^24^ During the study period, we had a stoma rate of 45% and a major complication rate of 16.7%. The stoma rate was 30% for low PCI (< 25) versus 81% for high PCI (> 25), which might be considered acceptable when weighted against the expected survival benefit of radical surgery. The criteria for stoma formation in our clinic are poor nutrition, hypoalbuminemia, multiple bowel resection, steroid usage, and other factors that increase the risk of anastomosis leakage. However, during and after the study period, we became more restrictive with stoma formation.

All the patients in our study with a PCI of 24 or lower had a complete CRS, with 88% experiencing minor complications. Complete CRS was reached for 67.3% of the patients with a PCI of 25–34, but 29.6% of this group had grade 3 or 4 complications, and 81.8% received a stoma. Consequently, patients with a PCI higher than 24 could benefit from neoadjuvant chemotherapy. However, the decision to perform surgery will never be based on carcinomatosis alone. Additional factors such as comorbidity, age, and the patient’s own will should be considered. Furthermore, the question of patient selection for NACT or PDS should be answered by randomized controlled trials such as the forthcoming Trial of Radical Upfront Surgical Therapy (TRUST).^25^

Attempts have been made to use PCI to predict operability before opting for CRS. Most studies have focused on imaging, whereas Llueca et al.^21^ have suggested a combination of CT scan and laparoscopy. A laparoscopy scoring system has been introduced,^26^ but the most crucial areas for resectability may be difficult to access with laparoscopy, resulting in hidden areas of carcinomatosis,^27^ making it less feasible. A recent Cochrane review^28^ concluded that laparoscopy could be a useful tool for identifying women with nonresectable disease, but the reviewed studies included women who had incomplete surgery, with laparoscopy predicting total resectability. Moreover, in recent years, CRS has become more extensive, making evaluation with laparoscopy more difficult. The usual limitations for laparoscopy are the presence of obstructing omental cake and adhesions, whereas a tear in the (often fragile) bowel during dissection might be hazardous. Laparoscopy also is connected with the risk of abdominal wall metastasis and disease upstaging.^29^

Imaging studies have shown that diffusion MRI is superior to CT scan because it provides greater accuracy, whereas CT underestimates small carcinomatosis nodules. However further studies are needed before preoperative patient selection is based on PCI, and our group has an ongoing study on this issue.

In conclusion, the PCI proved to be an excellent predictor of incomplete CRS, and more complications were found with a greater tumor burden. The PCI cutoff of 24 or higher was related to incomplete CRS and inoperability, and we suggest that with this PCI cutoff, neoadjuvant chemotherapy can be considered, especially for fragile patients. Our findings support further studies on PCI in preoperative imaging because more accurate knowledge of tumor load could enhance patient selection before surgery. We recommend that the PCI be used as a standard parameter in clinical management of advanced gynecologic cancers and included as a parameter in registers and research.

## Electronic supplementary material

Below is the link to the electronic supplementary material.

Supplementary material 1 (DOCX 154 kb)
