# Supplementary material for: The Peritoneal Cancer Index is a Strong Predictor of Incomplete Cytoreductive Surgery in Ovarian Cancer
Source: Ann Surg Oncol. 2020 May 29;28(1):244–51. doi: 10.1245/s10434-020-08649-6 (PMC7752870; doi:10.1245/s10434-020-08649-6)
Supplement: Supplementary file 1 — Supplementary material 1 (DOCX 154 kb) [file 10434_2020_8649_MOESM1_ESM.docx]

**Supplementary data:**

**S1: Sugarbaker's scheme for the determination of the peritoneal cancer index (PCI):**


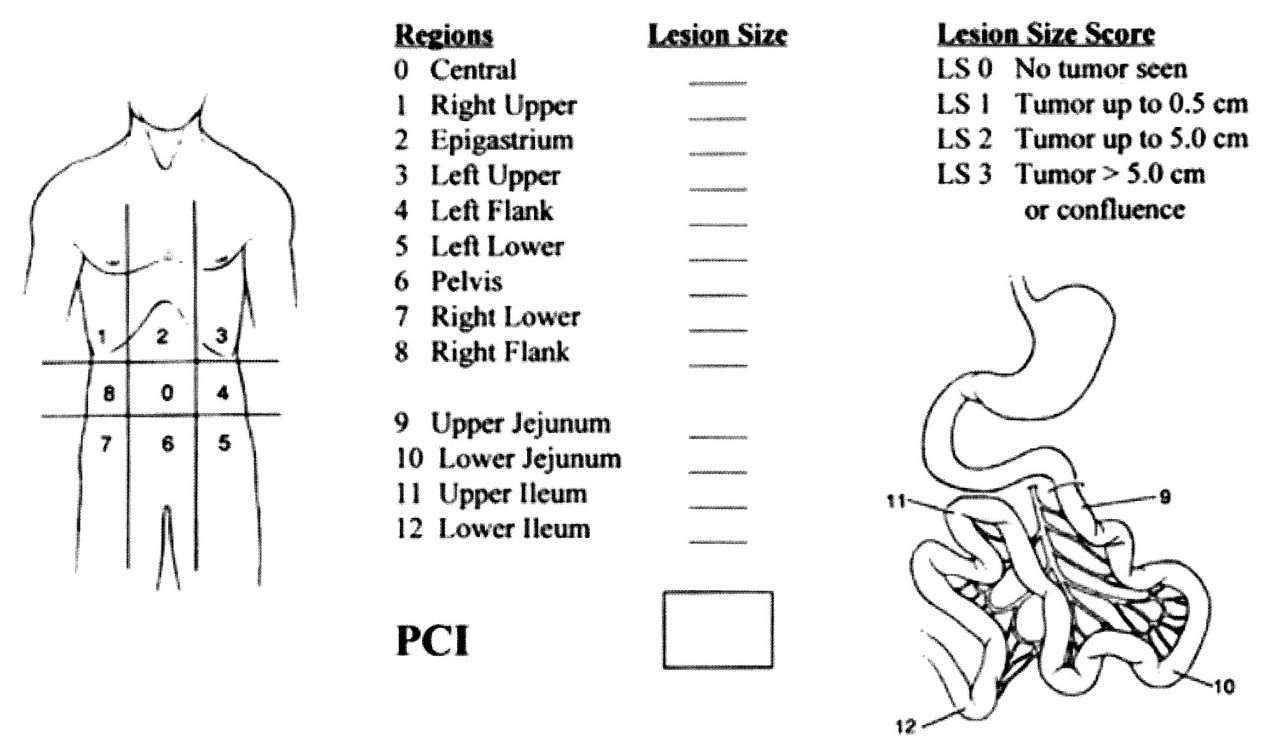


**S2: Peritoneal cancer index vs incomplete CRS, coordinates of the Receiver Operator Curve:**

|  | | |
| --- | --- | --- |
| Test Result Variable(s): PCI | | |
| Positive if Greater Than or Equal To^a^ | Sensitivity | 1 - Specificity |
| -1.00 | 1.000 | 1.000 |
| .50 | 1.000 | .975 |
| 1.50 | 1.000 | .969 |
| 2.50 | 1.000 | .963 |
| 3.50 | 1.000 | .913 |
| 4.50 | 1.000 | .906 |
| 5.50 | 1.000 | .881 |
| 6.50 | 1.000 | .856 |
| 7.50 | 1.000 | .844 |
| 8.50 | 1.000 | .813 |
| 9.50 | 1.000 | .763 |
| 10.50 | 1.000 | .744 |
| 11.50 | 1.000 | .719 |
| 12.50 | 1.000 | .644 |
| 13.50 | 1.000 | .613 |
| 14.50 | 1.000 | .588 |
| 15.50 | 1.000 | .575 |
| 16.50 | 1.000 | .538 |
| 17.50 | 1.000 | .519 |
| 18.50 | 1.000 | .475 |
| 19.50 | 1.000 | .438 |
| 20.50 | 1.000 | .425 |
| 21.50 | 1.000 | .400 |
| 22.50 | 1.000 | .363 |
| 23.50 | 1.000 | .319 |
| 24.50 | .950 | .244 |
| 25.50 | .900 | .200 |
| 26.50 | .900 | .175 |
| 27.50 | .850 | .138 |
| 28.50 | .800 | .113 |
| 29.50 | .750 | .088 |
| 31.00 | .550 | .063 |
| 32.50 | .500 | .025 |
| 33.50 | .450 | .006 |
| 34.50 | .300 | .000 |
| 35.50 | .200 | .000 |
| 36.50 | .050 | .000 |
| 38.00 | .000 | .000 |

**S3: B Inoperable patients, their PCI and description of failure site.**

| **Patient** | **PCI** | **Failure site:** | **Description:** |
| --- | --- | --- | --- |
| 1 | 30 | Small bowel | 1cm tumor nodules spread over the small bowel mesothelium and on the small bowel wall. No part of the small bowel without tumor spread. |
| 2 | 37 | Small bowel | Maximal 50-60cm of normal small bowel (in the proximal jejunum). |
| 3 | 34 | Small bowel | Tumor nodules on the whole length of the wall of the small bowel and the small bowel mesentery. |
| 4 | 30 | Small bowel | Carcinomatosis on the small bowel near ventricle, duodenum and liver hilum. |
| 5 | 30 | Small bowel | Massive carcinomatosis on all peritoneal surfaces. Maximal 10cm normal small bowel. |
| 6 | 29 | Small bowel | Carcinomatosis on the small bowel and transverse and descending colon.  Normal small bowel about 2m. |
| 7 | 27 | Small bowel | No small bowel without carcinomatosis. High tumor load in liver hilum, gallbladder and upper part of the abdomen. |
| 8 | 33 | Small bowel | Carcinomatosis on the whole length of ileum. Less on jejunum but maximal 2m normal small bowel. |
| 9 | 32 | Small bowel | Omental cake 12x4cm on colon transversus. Maximal 1.5m normal small bowel. |
| 10 | 35 | Small bowel | Massive carcinomatosis on the whole length of the small bowel mesentery. |
| 11 | 30 | Small bowel | Tumor nodules up to 1cm on the small bowel, no normal section. |
| 12 | 34 | Small bowel | Carcinomatosis on the small bowel mesentery. |
| 13 | 36 | Small bowel | Invasive tumor nodules with a diameter of 5-7mm on the small bowel,  maximal 3cm interval without carcinomatosis. |
| 14 | 30 | Small bowel | Carcinomatosis on the whole length of the small bowel. |
